# Higher attentional costs for numerosity estimation at high densities

**DOI:** 10.3758/s13414-019-01831-3

**Published:** 2019-08-12

**Authors:** Antonella Pomè, Giovanni Anobile, Guido Marco Cicchini, Aurora Scabia, David Charles Burr

**Affiliations:** 1 Department of Neuroscience, Psychology, Pharmacology and Child Health, University of Florence, Florence, Italy; 2 Department of Developmental Neuroscience, IRCCS Stella Maris Scientific Institute, Pisa, Italy; 3 Institute of Neuroscience, National Research Council, Pisa, Italy; 4 School of Psychology, University of Sydney, Camperdown, Sydney, Australia

**Keywords:** Attention, Dual-task performance

## Abstract

Humans can estimate numerosity over a large range, but the precision with which they do so varies considerably over that range. For very small sets, within the *subitizing* range of up to about four items, estimation is rapid and errorless. For intermediate numerosities, errors vary directly with the numerosity, following Weber’s law, but for very high numerosities, with very dense patterns, thresholds continue to rise with the square root of numerosity. This suggests that three different mechanisms operate over the number range. In this study we provide further evidence for three distinct numerosity mechanisms, by studying their dependence on attentional resources. We measured discrimination thresholds over a wide range of numerosities, while manipulating attentional load with both visual and auditory dual tasks. The results show that attentional effects on thresholds vary over the number range. Both visual and auditory attentional loads strongly affect subitizing, much more than for larger numerosities. Attentional costs remain stable over the estimation range, then rise again for very dense patterns. These results reinforce the idea that numerosity is processed by three separates but probably overlapping systems.

Humans can estimate the numerosity of large sets of items, usually with some error. However, for small sets up to about four, items can be estimated quickly and without error. This was first observed by Jevons (1871), and subsequently was termed *subitizing* by Kaufman and Lord (1949). Jevons also observed that after four items errors (in estimating the number of beans in a dish) increased in direct proportion to the number of beans estimated. This is a clear example of Weber’s law, amply confirmed by subsequent reports (Dehaene, 2011; Ross, 2003; Whalen, Gallistel, & Gelman, 1999). Besides this classical dichotomy, more recent evidence points to the existence of a third mechanism coming into play when judging numerosity at high densities, which might be linked to the perception of texture density. This third system is thought to be activated when visual items are highly packed and difficult to segregate spatially (Anobile, Cicchini, & Burr, 2014). In this range, the limiting factor appears to be not so much the absolute number of items, but their relative center-to-center distance (sparsity), as well as their viewing eccentricity (Anobile et al., 2014; Anobile, Turi, Cicchini, & Burr, 2015).

There is now good evidence that small (subitizable) sets of items activate separate processes. Evidence for subitizing comes largely from a discontinuity in reaction times, response variability, and accuracy. These parameters are consistently lower for numbers of 1 to 4, with performance sharply declining for larger numbers outside the subitizing range (Atkinson, Campbell, & Francis, 1976; Choo & Franconeri, 2014; Mandler & Shebo, 1982; Revkin, Piazza, Izard, Cohen, & Dehaene, 2008). Another method to differentiate between systems is to measure manipulations such as attention. Following this rationale, it has been shown that depriving visual attentional resources leads to massive detrimental effects of performance thresholds in the subitizing range, but far less for larger numbers (Burr, Turi, & Anobile, 2010; Egeth, Leonard, & Palomares, 2008; Olivers & Watson, 2008; Railo, Koivisto, Revonsuo, & Hannula, 2008; Vetter, Butterworth, & Bahrami, 2008). The same differential effects of attentional load have been detected cross-modally: Visual subitizing suffers greatly from both auditory and haptic distractors, whereas the estimation range is affected very little (Anobile, Turi, Cicchini, & Burr, 2012). Similarly, visual subitizing, but not estimation of larger numerosities, has been shown to be strongly impaired by concurrent visual working memory load (Piazza, Fumarola, Chinello, & Melcher, 2011). These results have been interpreted as a signature of partially independent systems for the subitizing and estimation regimes.

Many studies have investigated performance differences between the estimation range (in which items can be clearly segregated) and higher densities, in which items are not segregable. Studies have shown clear differences in the psychophysical laws governing precision for relatively sparse as compared with packed dot patterns: For sparse patterns, the discrimination thresholds are higher and obey Weber’s law; at higher numerosities, they decrease with the square root of numerosity (for a review, see Anobile et al., 2014; Anobile et al., 2015).

Various experimental manipulations can differentially affect the perception of low and high densities. For example, connecting dot patterns with short lines reduces the perceived numerosity considerably (Franconeri, Bemis, & Alvarez, 2009; He, Zhang, Zhou, & Chen, 2009; He, Zhou, Zhou, He, & Chen, 2015). However, Anobile, Cicchini, Pomè, and Burr (2017) showed that the effect was much reduced, and even inverted, for densely packed stimuli. Other recent evidence reinforcing the notion of separate mechanisms for sparse and dense stimuli comes from psychophysical studies pointing to different receptive-field sizes (Zimmermann, 2018), and from an electroencephalographic study showing different neural signatures (Fornaciai & Park, 2017). Differences in reaction times also point to three numerosity regimes (Pomè, Anobile, Cicchini, & Burr, 2019).

In the present study, we investigated the effects of visual and auditory attentional load on visual estimation of numerosities, over a wide range. The results are consistent with the existence of three regimes of number perception.

## Method

### Participants

Seven participants (five females, two males; mean age = 26 years, *SD* = 2.08) with normal or corrected-to-normal vision were tested on the visual spatial attention task; five of these were also tested on the auditory time bisection task (two did not give consent for the whole protocol). All participants performed the single-task control. All participants gave written informed consent, and the experimental procedures were approved by the local ethics committee (Comitato Etico Pediatrico Regionale—Azienda Ospedaliero-Universitaria Meyer—Firenze).

### Apparatus and stimuli

The experiment was run in a dimly lit room with stimuli presented on a 13-in. Macintosh monitor with 1,440 × 900 resolution at a 60-Hz refresh rate, mean luminance 60 cd/m^2^. Participants viewed the stimuli binocularly at a distance of 57 cm from the screen. The stimuli were generated and presented under Matlab 9.1 using PsychToolbox routines.

The stimuli for the numerosity task were two dot clouds of 6° diameter centered 10° right and lefts of a central fixation point. Each dot was positioned pseudorandomly within the dot cloud, with the condition that two dots (center to center) could not be separated by less than 0.25°. In a particular session, one cloud of dots (the reference, randomly right or left) maintained a particular numerosity across trials, whereas the other (the probe) varied around this numerosity. The number of dots in the probe patch varied according to the QUEST adaptive algorithm (Watson & Pelli, 1983), perturbed with Gaussian noise with a standard deviation 0.15 log units. In separate blocks, 14 different reference numerosities were tested: 3, 6, 8, 12, 18, 24, 32, 50, 64, 75, 100, 125, 150, or 200. The probe numerosities were curtailed to be within 1 and 600.

**Fig. 1:**
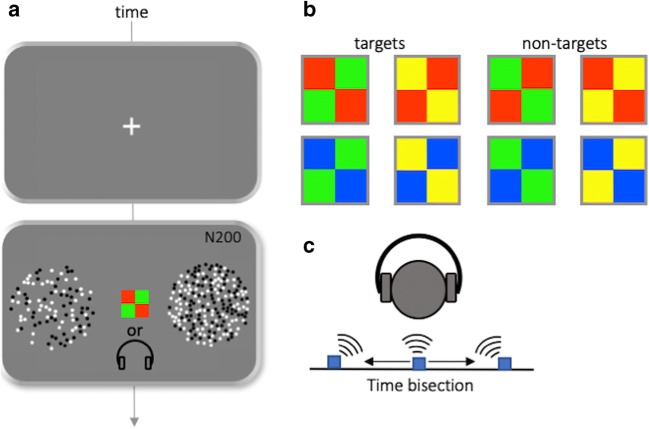
Perceived numerosity for our cross-modal attention experiment. (**a**) Each trial started with a fixation point, followed by two dot clouds presented together with the distractor. Both types of stimuli lasted for 500 ms. In the dual-task condition, participants responded first to the distractor task and then indicated which of the two clouds of dots seemed more numerous. In the single task, they performed only the numerosity task. (**b**) Conjunction stimuli displayed in the center of the screen for the visual distractor task. The stimulus was a target if it satisfied a specific conjunction of colors and orientations (see the Apparatus and Stimuli section for details). (**c**) Time bisection judgment in the auditory distractor condition. Participants were asked to perform an interval discrimination task, judging whether the middle tone was closer in time to the first or the third tone

The dot stimuli were presented for 500 ms, simultaneously with a visual or auditory distractor. The visual distractors (Fig. 1b) comprised four centrally positioned colored squares (3° × 3°), which could have eight color arrangements. The stimulus was a target if a specific conjunction of color and spatial arrangement was satisfied: two green squares along the right diagonal, or two yellow squares along the left diagonal. The auditory interval discrimination task was an interval bisection task with three 1300-Hz, 10-ms tones. The first and third were always played at 0 and 250 ms, and the second had a variable interval (60, 80, 90, 110, 120, or 140 ms). Participants were asked to report (by appropriate keypress) whether the second tone was temporally closer to the first or third tone.

### Procedure

In the single-task condition, participants were told to ignore the central distractor task and to indicate which of the two peripheral dot clouds contained more dots. In the dual-task conditions, participants first responded to the distractor task and then indicated which of the two arrays was more numerous. The order of tasks was pseudorandom across participants.

Before starting the experimental condition, all participants performed 30 training trials, in which they were asked to judge whether or not the central colored square was a target for the visual spatial attention task, or to report whether the second tone was temporally closer to the first or the third tone for the auditory time bisection task (if 75% accuracy was not attained, the session was repeated). In the main experiment, all trials started with a fixation point presented until the participant pressed a key to start the experiment, and then the primary and secondary stimuli were presented for 500 ms. Participants were tested with 14 different reference numerosity levels. The order with which each numerosity was tested was pseudorandom across participants and attentional conditions.

Three sessions of 30 trials each were run for each numerosity level and each attentional condition, yielding a psychometric function for that condition. The function was plotted and inspected visually, to ensure that it was monotonically ascending and well behaved. We also checked the estimate of the standard error of the mean: If this was greater than 30% of the estimated just-noticeable difference (JND), we added another session of 30 trials. In practice this happened on only 4% of the psychometric functions. On average, each participant had 1,260 trials.

### Data analyses

For each participant, the proportion of trials in which the probe appeared more numerous than the reference was plotted against the number of reference dots on a logarithmic scale and was fit with a cumulative Gaussian error function. The median (the numerosity corresponding to 50% left responses) gave the point of subjective equality (PSE), and the difference in numerosity required to pass from 50% to 75% correct responses defined the JND, a measure of precision. The JND divided by the reference numerosity yields the coefficient of variation (CV), a dimensionless index of precision that allows comparison of performance across numerosities. Where performance was errorless (as often occurred in the subitizing range in the single task), the JND was arbitrarily assigned as 0.001 dots.

Biases in PSE were tested by a series of Wilcoxon signed-rank tests (two-tailed) comparing, separately for each numerosity (14 levels) and attentional condition, the PSE shifts from the physical reference numerosity. The alpha level was Bonferroni corrected according to .05/14 (.0035).

To model numerosity-dependent changes in thresholds, CV-versus-numerosity curves above the subitizing range (*N* ≥ 6) were fitted with two-segment piecewise linear fits, with slope of the first segment set to zero and the second left free to vary. Standard error estimates for all fit parameters were obtained by bootstrap resampling of participants (10,000 reiterations) and fitting the data to the average group performance. The same iterations were used to calculate bootstrap sign-test *p* values. Residuals of the two-segment function (three parameters, baseline, knee point, and high-numerosity slope) were compared to those of a simple linear fit (two parameters) by means of the Akaike information criterion (AIC). By definition, the AIC of each model is

1 $$ AIC=2k-2\ln \left(\mathcal{L}\right) $$

where $\mathcal{L}$ is the maximal of the log-likelihood function and *k* is the number parameters in the model. The maximal of log-likelihood can be derived from the residual sum of squares according to the following formula:

2 $$ \mathcal{L}=-\frac{n}{2}\ln \left( RSS/n\right)+C $$

where *RSS* is the residual sum of squares, *n* is the number of data points, and *C* is a constant that depends solely on the data and does not vary from model to model. Overall, save for a common constant term *C* + ln(*n*), the AIC of a model is

3 $$ AIC=2k+n\ln (RSS) $$

The attentional cost was measured for each individual as the ratio between CVs in the single- and dual-task conditions. The statistical significance of the attentional cost within the numerosity range was measured by bootstrap sign test (BST) by resampling (10,000 times, with replacement) participants and numerosities within the range (except for the subitizing range, where only one numerosity was tested). The proportion of times in which the cost was less than or equal to unity (null hypothesis) was taken as the BST *p* value.

The differential attentional cost between numerosity regimes was also measured by a similar procedure to yield average CVs for each numerosity range, which were then pitted against each other. By convention, the reported *p* values represent the proportions of times the attentional cost of the estimation regime exceeded that in the other regime (10,000 iterations).

### Sample size

To determine the appropriate sample size, we ran two bootstrap power analyses for the two analyses of attentional costs. The first is a comparison of CVs of the single and dual tasks within one numerosity regime. To mirror our paradigm, we assumed each participant would be tested over a broad range of numerosities with a psychometric curve based on 90 two-alternative forced choice trials at each numerosity. Given the previous literature and the present choice of reference numerosities, it was reasonable to assume that at least three would fall in one regime and three into the other. Thus, conservatively, we assumed that the measure of attentional costs within one regime would be based on the average CVs in three psychometric curves in the single and dual tasks. Population variance was derived from the previous literature (Burr et al., 2010; Tibber, Greenwood, & Dakin, 2012) and was assumed to be 20%. Finally, we assumed that, to be detected, attentional costs would have to be of a factor of 1.2 (less than half of the effect documented by Burr et al., 2010). Simulations demonstrated that a sample size of four participants would be sufficient to return a true positive on 91% of the cases.

In the second power analysis, we applied similar reasoning to a comparison between the attentional costs across regimes. We assumed the attentional costs in the two regimes might differ by 25%, since a smaller difference would be of little importance. Simulations showed that four participants were sufficient to detect such a difference with a power of 94%. Hence, a sample size of five was deemed appropriate to address the experimental questions posed in the study. Nevertheless, because replicability is important, we ran an addition study to replicate our main results, with an additional nine naïve participants.

## Results

We tested the effect of attentional load on numerosity perception over a wide range of numerosities. We first examined whether the attentional manipulations affected PSEs. We found no significant deviation from the physical reference numerosity (all *p*s > .01, two tailed *Z* tests, corrected *α* = .05/13 = ~ .004). However, this was to be expected, since the probe and reference stimuli were randomized in position.

**Fig. 2:**
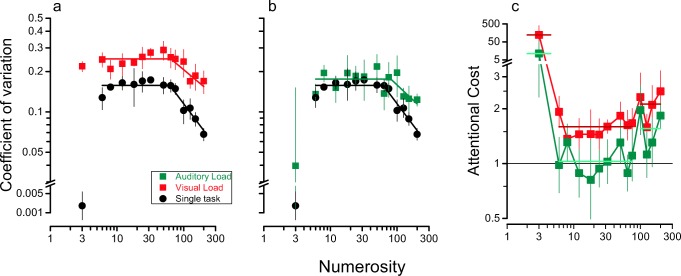
Precision and attentional cost for both visual and auditory load. (**a**, **b**) Mean coefficients of variation (CV; the just-noticeable difference normalized by numerosity) as a function of target number for the single task and the distractor conditions (**a**, visual; **b**, auditory). Visual attentional load strongly impairs precision in the subitizing range (4 and below), and also in the density perception range (from 100 dots); a smaller but similar effect occurs for the auditory load condition. (**c**) Attentional costs (precision in the dual-task condition divided by the precision in the single-task condition). Numerosity precision was more affected by visual than by auditory load. The discontinuous horizontal lines show the means per range for both conditions, showing the mean over that range: subitizing (up to 3), estimation (up to ~ 80), and texture density (up to 200)

We then looked at sensory thresholds. Figure 2a and b plot average normalized discrimination thresholds (CVs), separately for the two attentional conditions (visual and auditory), as a function of dot numerosity. The curves passing through the data were two-segment piecewise linear fits that excluded the subitizing range (≥ 6), the first curve of slope zero and the second left free to vary. For the single-task condition, the CV was near zero in the subitizing range and then rose to about 0.18 for numbers above 6, remaining constant over the estimation range. For numerosities higher than 60, CVs decreased steadily with numerosity, with log–log slopes of – 0.65 ± 0.07. The two-limbed function fitted the data better than a single linear function (taking into account the degrees of freedom), in both log–log (AIC – 42 vs. – 15, fit residuals 0.02 L.U. vs. 0.223 L.U.) and lin–lin (AIC – 69 vs. – 56, residuals 0.0036 vs. 0.0099) coordinates. This reinforced the idea of two separate psychophysical regimes.

The precision for the two attentional conditions also followed a two-limbed function, with log–log slopes of – 0.47 ± 0.07 and – 0.65 ± 0.17. Interestingly, the knee points for the two conditions (64 ± 15 and 81 ± 16 for visual and auditory) fell close to that of the single-task condition (statistically indistinguishable, all *p* values > .1), indicating that the boundaries of the three regimes were similar in the two conditions.

We calculated the visual and auditory attentional costs as the ratio of the dual to single CVs (Fig. 2c). At low numerosities (*N* < 6), the visual dual-task raised the CV from ~ 0 to 0.22, a factor of 121 (BST *p* < .001), and the auditory task raised the CV by a factor of 11.2 (from ~ 0 to .039, BST *p* = .018). In the estimation range (6 < *N* < 60) the visual dual task had less effect than in the subitizing range, raising CVs from 0.16 to 0.25 (a factor of 1.6, BST *p* < .001). The auditory dual task had a negligible impact on CVs in this range (factor of 1.02, BST *p* = .5). In the texture density regime (*N* > 75), attentional costs rose again (visual dual task, factor of 2, BST *p* < .001; auditory dual task, factor of 1.58, BST *p* = .036).

A bootstrap *t* test of attentional costs revealed that the effects of the dual tasks in the three regimes were different from each other. In particular, the costs in the estimation and density regimes differed for both the visual distractor (*p* = .037) and the auditory distractor (*p* = .005). The attentional cost in the subitizing range was also markedly higher than in the estimation range (*p* = .0006, visual distractor; *p* = .047, auditory distractor).

To verify that the differences in attentional costs between ranges did not result from a change in the resources allocated to the primary task, we calculated the average accuracy in the three regimes for both types of distractors. Performance in the distractor visual task was 92%, 96%, and 96.2%, respectively, for subitizing, estimation, and density perception, and 98%, 97%, and 97% for the three regimes with the auditory distractors. Bootstrap *t* tests revealed that none of these were statistically significant (all *p*s > .15).

### Replication

Replicability is important. We therefore ran a replication study on nine new, naïve participants to verify the main results of this study: that attentional costs were different for the three regimes of numerosity perception. We tested three sample numerosities, representative of the subitizing, estimation, and texture ranges: 3, 24, and 150.

**Fig. 3:**
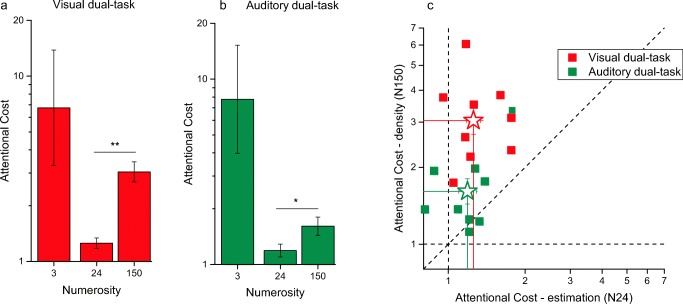
Replication study. (**a**, **b**) Geometric averages of attentional costs (ratio of dual-task to single-task thresholds) measured for nine new participants for three representative target numbers (3 for subitizing, 24 for the estimation range, and 150 for the density range). As before, attentional loads strongly impaired precision in the subitizing range (3) for both visual and auditory distractors. Importantly, the cost in the density range (150) was greater than that for the estimation range (24). (**c**) Attentional costs in the density range plotted against those for the estimation range, for all nine participants (square symbols). Stars show the geometric means. For the visual dual task, all nine participants showed higher attentional costs in the texture than in the estimation range; the costs of the auditory task for seven out of the nine participants were greater in the texture condition. Stars show significance (one-tailed paired *t* tests): ^*^p < .05; ^**^p < .01

Figure 3 shows that this supplementary study completely replicated the main result. For the visual distractor (Fig. 3a), the greatest cost was in the subitizing range, by a factor of 6.75, supporting this and previous research (Anobile et al., 2012; Burr, Anobile, & Turi, 2011; Burr et al., 2010).

Similarly, the attentional cost in the texture range was more than twice that in the estimation range, a factor of 3.04 compared to 1.25. This difference was highly significant [one-tailed *t* test: *t*(8) = 6.278, *p* = .0013]. The trend of the results with the auditory distractor (Fig. 3b) was similar, although the effects were weaker. The attentional cost was highest for subitizing (7.8), and higher for texture than for estimation (1.6 and 1.19, respectively). The difference between texture and estimation, although smaller than that for vision, remained significant [*t*(8) = 2.89, *p* = .015].

Figure 3c shows the individual results. For all nine participants, the attentional cost of the visual task was higher in the texture than in the estimation range; the cost of the auditory task was in general much less, but for seven out of nine participants it was greater in the texture condition. Thus, the trend of the main results was amply confirmed on replication.

## Discussion

Three separate regimes have been proposed for numerosity perception: subitizing, estimation, and texture density (for reviews, see Anobile, Cicchini, & Burr, 2016; Burr, Anobile, & Arrighi, 2017). Here we have provided further evidence for separate mechanisms underpinning these three regimes, by investigating the roles of visual and auditory attentional resources on discrimination thresholds over these ranges.

We first replicated our earlier study showing different psychophysical laws for thresholds in the three regimes. In the baseline condition, as expected, discrimination thresholds were near zero in the subitizing range, obeyed Weber’s law for intermediate numerosities, and then decreased according to a square root law for denser stimuli. Attentional load completely changed this pattern of results. As was previously shown for magnitude estimation tasks, attentional load greatly affected the subitizing range, to the extent that thresholds became similar to those in the estimation range (Burr et al., 2010), implying the existence of two separate but partially overlapping systems: estimation mechanisms, which probably extend into the subitizing range (Burr et al., 2011), supplemented by the attention-dependent subitizing system. When subitizing is compromised by depriving it of attention, estimation remains possible and yields CVs similar to those in the estimation range.

Attentional load (visual and auditory) had a greater effect on subitizing than on estimation, and increased again at higher densities. Numerosities higher than 60–80 dots were more affected by attentional load (both visual and auditory) than were lower (nonsubitizing) numerosities. This major result was confirmed on a replication of key numerosities with an additional nine naïve participants. These results reinforce suggestions of a third regime of numerosity perception. It is interesting that the mechanism that suffered least from depriving it of attentional resources was the “estimation range,” which suffered only a slight cost with the visual task, and no cost at all with the auditory task. Given that the two distractor tasks were different in nature (visuospatial vs. auditory–temporal), we cannot directly compare the modality-specific costs with each other. However, it is interesting that these diverse distractors led to qualitatively similar relative effects on thresholds over the three ranges.

There is now a better understanding of the involvement of attentional and visual working memory in the judgment of numerosities within the subitizing range (Anobile et al., 2012; Burr et al., 2011; Burr et al., 2010; Knops, Piazza, Sengupta, Eger, & Melcher, 2014; Piazza et al., 2011; Vetter et al., 2008; Vetter, Butterworth, & Bahrami, 2011). But why do judgments of very high numerosities (density regime) require more attentional resources than do intermediate (estimation regime) numerosities? We previously demonstrated that for tightly packed stimuli, the number of items is not perceived directly, but stimulus density (e.g., interdot distance) dominates judgments (Anobile, Castaldi, Turi, Tinelli, & Burr, 2016; Anobile et al., 2014; Burr et al., 2017; Cicchini, Anobile, & Burr, 2016). Other studies have shown that texture segregation and discrimination tasks require attentional resources (Landy & Graham, 2004; Yeshurun & Carrasco, 2000). Indeed, Tibber et al. (2012) found profound attentional costs in a dot-array density comparison task. Together, these results suggest that numerosity judgments for dense patterns require more attentional resources than those for sparse stimuli, because they tap an attention-dependent system that encodes texture density rather than numerosity. It has been shown that primary sensory attributes are robust to cross-modal attentional interference (Alais, Morrone, & Burr, 2006). Our results are consistent with this, and further they support the notion that number estimation is a primary visual attribute that is extracted spontaneously from the visual scene, at least for intermediate numerosities (Cicchini et al., 2016), without heavy recourse to attentional resources.

The discontinuity in psychophysical performance between estimation and texture density does not necessarily imply the existence of three totally independent systems. It is possible, indeed probable, that estimation mechanisms operate over the entire range, but that this system is supplemented by attentional mechanisms at low and very high numerosities. There is good evidence for an attention-dependent subitizing mechanism in the low range, allowing for perfect enumeration; but when attention is drawn from this mechanism by dual tasks, the estimation system continues to operate (Burr et al., 2011). The same interchange may occur at the high range: Texture mechanisms may normally operate on local texture, but when these are impaired, estimation mechanisms could take over. The numerosity system thus may always be active, but not always called into play. Since numerosity thresholds for sparse but not for dense stimuli are correlated with math abilities (Anobile et al., 2016), it would be interesting to test whether the correlation would also emerge for the discrimination of dense stimuli under attentional load.
